# Workplace exposure to UV radiation and strategies to minimize cancer risk

**DOI:** 10.1093/bmb/ldac019

**Published:** 2022-08-17

**Authors:** J W Cherrie, M P C Cherrie

**Affiliations:** IOM, Research Avenue North, Edinburgh EH14 4AP, UK; Institute of Biological Chemistry, Biophysics and Bioengineering, Heriot Watt University, Edinburgh EH14 4AS, UK; IOM, Research Avenue North, Edinburgh EH14 4AP, UK

**Keywords:** ultraviolet radiation, sunlight, occupational exposure, outdoor work

## Abstract

**Background:**

Workplace exposure to solar ultraviolet (UV) causes malignant melanoma and non-melanoma skin cancer. The evidence for beneficial effects of solar UV exposure in reducing the risks for other cancers is increasing. The intensity of UV radiation at the Earth’s surface is dependent on latitude, but even in northern European countries exposure can be high enough for outdoor work to cause skin cancer.

**Growing points:**

Awareness of the health risks and benefits of occupational solar UV exposure is poor. Actions to reduce the risk of skin cancer have been identified and employers should recognize their responsibility to actively manage these risks. There is evidence for reduced risks for breast, ovarian and colorectal cancer and possibly other cancers linked to solar UV exposure.

**Sources of data:**

This narrative review draws on published scientific articles and material designed to assist identifying strategies to protect workers from solar UV exposure.

**Areas of agreement:**

Solar UV exposure can be harmful. Wavelengths in the UVB range are more effective in causing erythema and DNA damage. Solar UV is the main source of vitamin D for most people. Primary and secondary prevention for skin cancer can potentially eliminate these risks but the evidence for effectiveness is limited.

**Areas of controversy:**

Potential health benefits of UV exposure, particularly for reduced cancer risk. Determining and communicating optimal exposure to maximize health benefits. The risk of non-melanoma skin cancers may be more than doubled for some workers in temperate latitudes.

**Areas timely for developing research:**

Exposure-response epidemiological studies; studies of the health benefits of occupational UV exposure; studies of the effectiveness of intervention strategies to prevent skin cancer. Use of low-cost UV sensors in workplaces.

## Background

Ultraviolet (UV) radiation is an invisible part of the electromagnetic spectrum with wavelengths shorter than those of visible light. The main source of UV exposure is sunlight, although some industrial processes, such as electric arc welding, may also emit anthropogenic UV radiation. We conventionally divide the UV spectrum into three bands based on the interaction of the radiation with matter and human cells: UVC (100–280 nm wavelength) is completely absorbed in the Earth’s stratosphere by ozone and so is very rarely a hazard to health (other than through accidental exposure from artificial UV sources^1^); UVB (280–315 nm) is also absorbed in the atmosphere, but around 5% penetrates to ground level and damages the upper level of the epidermis due to its short wavelength; and UVA (315–400 nm) is poorly absorbed in the atmosphere and penetrates more deeply into skin, reaching collagen fibres and this is why UVA exposure causes skin ageing.^2^ The ratio of UVA to UVB in incoming solar irradiance is around 20:1, although this varies with latitude, season, time of the day and atmospheric conditions such as cloud cover, given that these attenuate UVB more than UVA.^3^

For most people UV radiation is the main source of vitamin D, although diet and supplements also contribute to circulating vitamin D (25-hydroxyvitamin D or 25(OH)D).^4^ Vitamin D status tends to have a strong seasonal pattern, especially at high latitudes; in winter, around half of the British population will have 25(OH)D levels below 40 nmol/l (1 definition of insufficiency).^5^ UV, particularly UVB, also has the potential to acutely cause erythema (slight reddening of skin) and then an adaptive increase in epidermal melanin in fair-skinned people. Furthermore, it has the potential to damage DNA, including causing mutations in the p53-gene, resulting in loss of its tumour suppression function and production of reactive oxygen species (ROS) associated with initiation, promotion and progression of skin cancer.^6^ Dark skin pigmentation is protective against skin damage, including DNA damage.

The International Agency for Research on Cancer (IARC) has classified solar radiation and UVA, UVB and UVC as carcinogenic to humans (Group 1), based on sufficient evidence in human epidemiology and data from experimental animal studies.^7^ It was concluded that solar radiation causes cutaneous malignant melanoma, along with squamous cell carcinoma (SCC) and basal cell carcinoma (BCC) of the skin (non-melanoma skin cancer, NMSC). IARC also noted positive associations in the epidemiological evidence for exposure to solar radiation and cancer of the lip, conjunctival SSC and ocular melanoma. In addition, IARC classified welding, which can generate UV radiation, as a cause of ocular melanoma. Some rare melanomas, e.g. acral melanoma, are most likely not linked to UV exposure.^8^

Globally there are 7.7 million cases of NMSC and around 310 000 cases of malignant melanoma annually.^9^ The incidence and prevalence of both cancer groups have been increasing in many parts of the world over the past 25 years,^10^ although it is possible that NMSC may be underreported because of removal of lesions without histopathologic assessment.^11^ Associated Disability Adjusted Life Years lost are highest in ‘High Income’ countries and amongst countries in Central Europe, Eastern Europe and Central Asia. Peters *et al*.^12^ estimate that around 6% (4556 cases) of NMSC in Canada were attributable to occupational exposure to solar UV. Rushton *et al*.^13^ estimated around 2.3% of NMSC cases in Great Britain (1541 cases, 12 deaths) were attributable to work and, Ruston and Hutchings^14^ later estimated that 2% of incident cutaneous malignant melanomas (241 cases, 48 deaths) were work-related, although they accept the evidence for a causal association with workplace UV exposure was not entirely convincing. These occupational risks affect outdoor workers, mainly agricultural, construction, public administration and defence, and land transport workers.

The International Commission on Non-Ionizing Radiation Protection (ICNIRP) set a guideline limit for artificial UV exposure on unprotected skin and eyes of 30 J/m^2^ within an 8-h period.^15^ Effective irradiance (W/m^2^) is calculated by weighting spectral irradiance by the ICNIRP action spectrum, for each wavelength interval between 180 and 400 nm. This can be converted to the maximum permissible exposure duration by dividing the ICNIRP guideline (30 J/m^2^) by the effective irradiance, assuming irradiance is constant.

**Fig. 1:**
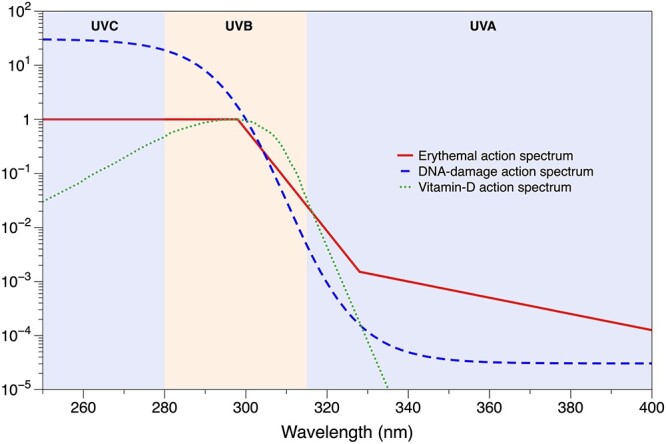
Action spectra for UV exposure (adapted from https://www.temis.nl/uvradiation/product/action.html).

Exposure is often measured in units of Standard Erythemal Dose (SED; 100 J/m^2^), to reflect the ability of the radiation to produce erythema 6–24 h after exposure and remain for 24 h thereafter. This is calculated by weighting spectral irradiance by the CIE erythemal action spectrum^16^ (Fig. 1). The ICNIRP guideline corresponds to between 1.0 and 1.33 SED per day.^17^ It is recognized that the guideline is conservative and the Minimal Erythemal Dose ranges from 2 SED for someone with fair skin (Fitzpatrick phototype I or II, Celtic) to 15 SED for someone with dark brown or black skin (Fitzpatrick VI); for these phototypes where the skin is adapted for sun exposure the corresponding values are 5 SED (phototype I or II) and 80 SED (phototype VI). Skin pigmentation and its consequences on the effects of UV exposure are discussed by Del Bino *et al*.^18^

The aim of this narrative review is to outline to non-specialist clinicians the best strategy to balance the detrimental and beneficial cancer risks for workers exposed to solar UV and to discuss controversial areas where the scientific and practice evidence is still emerging. We only focus on cancer, but there are other risks and benefits associated with UV exposure and in practice these also need consideration.^19^ The paper mostly discusses the issues in relation to European and North American workplaces. No new data were generated or analysed in support of this review.

## Workplace exposure to UV radiation and risk management

### Exposure measurement

UV irradiance can be accurately measured at fixed locations using radiometers. However, as occupational exposure is dependent on both the local environmental conditions and the behaviour of the workers, personal dosimeters are preferable. There are several devices available, mostly using two general approaches: either photosensitive plastic films that change their optical properties when exposed to UV (chemical UV dosimeters) or semiconductor-based electronic sensors (electronic UV dosimeters).^20^ The skin’s response to UV depends on the wavelength of the radiation with, for example, the erythemal response between 250 and 400 nm, with the peak response between 250–298 nm (UVB). The biologically relevant UV dose can be estimated by adjusting the measurements using a dimensionless ‘action spectrum’ weighting factor (Fig. 1).

Polysulphone (PSF) film is the commonest material used for chemical UV sensors, usually fabricated into a badge holder that can be worn by the worker.^20^ The film undergoes degradation on exposure to UV and this increases the absorbance, and the cumulative UV dose can be measured using pre and post measurement of absorbance of the PSF film with a spectrophotometer at 330 nm wavelength. The spectral response of PSF is similar to the erythemal action spectrum. Recently, chemical dosimeters based on photosensitive dyes have been developed (e.g. https://logicink.com/products/daily-suncare-signal), although these are generally only qualitative indicators of exposure.

Electronic dosimeters are generally based around a photodiode sensor with a spectral response designed to match the erythemal action spectrum, with the instantaneous sensor measurements being logged every few seconds. Data can then be downloaded after days or weeks of use and processed by computer. For example, Wittlich and colleagues used electronic dosimeters (the GENESIS-UV system) to undertake measurements of UV exposure for outdoor construction workers from several EU countries (latitude between 34 and 56° N).^21^ Research suggests that electronic dosimeters and PSF monitors systematically differ in assessment of UV exposure by 20–30%.^22^ There are also low-cost electronic dosimeters available for consumer use, although they have uncertain accuracy and are generally unsuitable for workplace assessments; all electronic dosimeters require calibration to fixed site ‘research-grade’ measurements to ensure the accuracy of the data.

Measurements of exposure have been undertaken in several northern latitude areas in Europe and North America, including in the UK. Grandahl *et al*.^23^ presented data for a range of Danish (latitude 56° N) outdoor workers measured between April and September. Median daily exposure in each month ranged from 1.6 to 2.4 SED, with exposures highest for occupations such as roofers, concrete workers and road workers, and lowest for unskilled labourers, dock workers and carpenters. Data for outdoor workers in Alberta, Canadian (latitude 54° N),^24^ showed a similar range of exposure depending on job (mean for occupations from 0.7 to 2.6 SED) with an overall daily mean of 1.9 SED. Cherrie *et al*.^25^ reported data for British outdoor construction workers with mean exposure of 2.0 SED (latitude 51.5–60° N). Data for Danish masons working outdoors (latitude 56° N) showed mean daily exposures over each month of 0.4–4.7 SED and an overall daily average of 3.2 SED.^26^ Corresponding data for German workers (latitude 47–55° N) showed slightly higher daily mean exposures over a year of 3.5 SED.

### Protective measures

There are no specific legal occupational exposure limits on solar UV exposure in any country around the world.^27^ Typical official advice to outdoor workers to control risks from solar UV includes (https://www.hse.gov.uk/skin/employ/sunprotect.htm):

- Wear long-sleeved top and long trousers;
- Wear a hat with a brim or a flap that covers the ears and neck;
- If possible, keep in the shade;
- Use sunscreen with a high protection factor.

Although these measures can undoubtedly reduce the solar UV exposure of workers using them,^17^ there is little evidence that they are consistently used. This is probably because they are impractical (unsafe or uncomfortable) or due to workplace culture.^28^ For example, Peters and colleagues^29^ questioned 77 outdoor workers in Canada and found that 79% often/always wore hats and 82% wore sleeved shirts, but only 8% consistently sought shade while working and 29% often/always wore sunscreen. In a systematic review of the scientific literature, Reinau and colleagues concluded that the use of sun-protection was essentially inadequate, with many workers reporting they never or rarely wore a long-sleeved shirt (50–80%), sun-protective hats (30–80%) and sunscreen (30–100%).^30^ Providing shaded areas for breaks and task rotation/flexibility during peak UVR hours may be more effective than relying on personal protective behaviours.^31^ There is also no good evidence as to the effectiveness of these approaches in preventing skin cancer. For example, Sanchez *et al*.^32^ reviewed the evidence from randomized controlled trials for the prevention of NMSC and found only a single uninformative study (because of potential bias). A systematic review and meta-analysis incorporating 29 studies (25 case–control, two cohort, one cross-sectional and one controlled trial, which was included in the review by Sanchez *et al*.) also found no evidence of protection against skin cancer from sunscreen use.^33^ Part of the reason for the apparent ineffectiveness of protective strategies for skin cancer may be the attitude of workers towards acquiring a suntan. For example, Miles *et al*.^34^ reported results from a survey of UK adults that showed two-thirds of respondents agreed that having a suntan made them look healthier and around half felt that a suntan made them more attractive. Cherrie *et al*.^25^ found similar results in their study of outdoor workers in Britain.

Improved information and guidance for workers using electronic media have been suggested as one way of addressing the difficulties in managing UV exposure of outdoor workers. Niu *et al*.^35^ carried out a systematic review of digital interventions for sun protection and skin self-examination, such as websites, mobile applications and text messaging. They showed that these interventions were equally or more effective than conventional interventions and more effective than not intervening. However, it is unlikely that this type of approach, on its own, would be sufficient to change long-term behaviour and attitudes of workers to sun exposure.

John and colleagues^27^ provide a Global Call to Action, launched in 2019 at the 1st Multi-Stakeholder Summit on Occupational Skin Cancer, to improve the legislation to protect outdoor workers from solar UV and to provide compensation for workers with NMSC, improve and standardize the reporting of NMSC and encourage employers to use tools to measure exposure to UV in the workplace as a perquisite for effective risk management.

## Understanding the risk of NMSC in relation to UV exposure and compensation for British workers with these diseases

It is accepted that outdoor workers in the UK, particularly those involved in farming, seafaring, and outdoor construction work, are at increased risk of NMSC, although it is not currently possible for workers in Britain to claim Industrial Injuries Disablement Benefit (IIDB) if they have NMSC caused by their work. The main rationale for this is the absence of consistent evidence relating risk to the duration of outdoor work in the sun from epidemiological studies carried out in the UK.^36^ Diseases can be prescribed under the IIDB scheme if Government ministers are satisfied that the disease can be caused by work, and it is reasonably certain that such a link can be made in an individual claimant’s circumstances. ‘Reasonably certain’ is taken to mean that it is more likely than not that the disease was caused by the work activities, and this has often been interpreted as the work being linked with a doubling of risk in an epidemiological study.

**Fig. 2:**
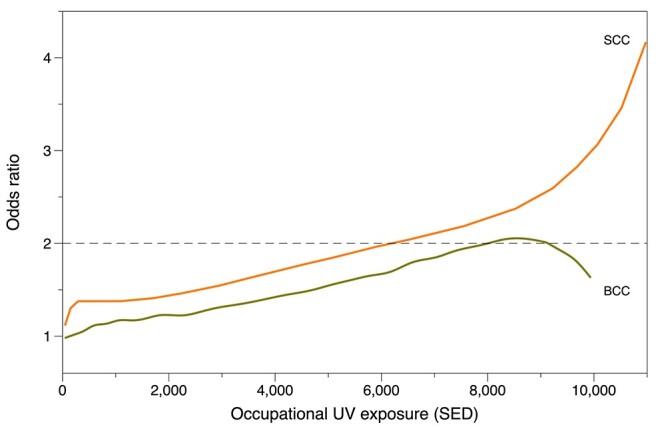
Exposure-response relationships for SCC^37^ and BCC.^38^

Recent research from Germany demonstrated clear exposure-response relationships for both SCC^37^ and BCC^38^ and exposure to UV from sunlight (Fig. 2 shows the relationships for occupational exposure). These researchers carried out two case–control studies identifying patients with a new cancer diagnosis drawn from eight dermatology clinics throughout Germany along with sex- and age-matched controls without the relevant cancer recruited from regional residents’ registration offices. Information about potential UV exposure throughout life, both at work and from leisure activities, was collected by questionnaire and UV exposure was estimated as SED using an algorithm based on measurements of UV.^39^ Further analysis of the BCC data showed that the risk was independent of tumour localization, histological subtype and skin colour (n.b. 97% of study participants had Fitzpatrick phototype III or less).^40^ The researchers found a doubling of the risk for SCC for a lifetime occupational exposure of 6348 SED, with the corresponding figure for BCC being 7945 SED; after adjusting for occupational exposure, non-occupational exposure was not significantly associated with risk for either cancer. In both cases these exposures were around the 90th percentile of occupational exposure for the subjects.

The presence of an exposure-response relationship provides an alternative approach to identify jobs where NMSC is more likely than not caused by workplace exposure and workers could therefore be compensated for their disease. If we assume the average exposure of an outdoor worker in Britain was 2 SED, as measured by Cherrie *et al*.^25^ amongst outdoor construction workers, on around 140 days each year (using the same assumptions as Schmitt and colleagues^37^^,^^38^ for work between April and October^21^), then British outdoor workers would on average have a doubled risk of BCC after 28 years and for SCC after 22 years.

There is no definitive list of jobs in Britain which involve outdoor work, although there are such data for jobs in the USA (https://www.onetonline.org) and it is plausible that these would be like British work. This O*NET database contains data obtained by questionnaire on descriptions of the work for use by job seekers and others. Information is available for US Standardized Occupational Codes for ‘Work Context - Outdoors, Exposed to Weather’ and, for example, there are 64 job titles where the workers on average are outside >90% of the time. Around a third of these jobs were in construction and a further third in transportation and installation, maintenance and repair jobs. However, the jobs were distributed through most of the main occupational groups, e.g. postal delivery workers in Office and Administrative Support Occupations, and not all jobs in some groups that might be expected to be at risk were included, e.g. only three of 13 job titles in farming and fishing were identified. Nevertheless, the list could form the basis of identifying jobs at risk of NMSC. In addition, there are Job-Exposure Matrices (JEMs) that have recently been developed to assess UV or visible light exposure in epidemiological studies that could further help identify specific British jobs where workers are at risk of NMSC^41–43^ and algorithm-based assessments of UV exposure.^44^

Further research, both on the magnitude of UV exposure and the duration of outdoor work for key occupations in Britain, would be advantageous, although as discussed a practicable way of ascribing jobs where workers are at risk of developing NMSC is available.

## Health benefits of UV exposure at work

### Summary of the epidemiological evidence

In recent years there has been increasing evidence for possible effects of UV exposure or vitamin D levels, a proxy for UV exposure, on cancers other than skin cancers. Epidemiological studies investigating these risks broadly fall into four categories: effects of supplementation with vitamin D and/or total vitamin D intake, associations with levels of circulating vitamin D pre-diagnosis, associations with estimated exposure to UV and Mendelian randomization (MR) studies. MR studies use single nucleotide polymorphisms (SNPs) at common sites (e.g. the vitamin D binding receptor and vitamin D binding protein) that are associated with lower circulating 25(OH)D concentrations. As these SNPs are presumed to be distributed at random across the population at conception, they act as instrumental variables that are not confounded by factors such as sex, ethnicity or socioeconomic status.

Following from an earlier analysis, Autier and colleagues^45^ published a systematic review of meta-analyses of vitamin D supplementation and non-skeletal disorders published between 2013 and 2017. They found a small reduction in all-cause mortality associated with daily intake of 10–20 μg (400–800 IU) of vitamin D and a larger and statistically significant reduction in **cancer mortality** (relative risk 0.88). However, there was no indication of a reduced risk of **cancer incidence**, and it has been suggested that the mortality results could be due to bias because of the high number of participants who dropped out of the trials before completion.^46^ More recent reviews and meta-analyses generally support the absence of any benefit of reduced cancer risk from vitamin D supplementation.^47^

Lopez-Caleya *et al*.^48^ carried out a review and meta-analysis of studies that had investigated total vitamin D intake (dietary and supplementation) and incidence of **colorectal cancer**, which showed a small statistically significant reduction in risk (odds ratio 0.96/100 IU/day of vitamin D, 95% confidence interval (95% CI) 0.93–0.98). MR studies have shown no evidence of any reduction in **colorectal cancer** risk with increased Vitamin D levels.^49^ There is no evidence that vitamin D supplementation is associated with a reduced risk of **breast cancer.**^50^ However, a meta-analysis of studies of circulating vitamin D and **breast cancer** showed a protective effect of 25(OH)D and breast cancer development in premenopausal women (odds ratio between the highest and lowest vitamin D categories 0.67, 95% CI 0.49–0.92).^51^ Hiller *et al*.^52^ carried out a systematic review and meta-analysis of epidemiological studies examining exposure to solar UV and breast cancer. They found a lower risk of **breast cancer** for women spending >1 h per day in the sun during summer over a lifetime or during adulthood compared with less than an hour each day (relative risk 0.84; 95% CI: 0.77–0.91), although there was no association with ambient UV intensity. A large prospective cohort study in the USA investigated incidence of **breast cancer** in association with UV exposure (expressed as mW/m^2^), categorized in quintiles.^53^ In this study, there was no association with the overall incidence of breast cancer, but higher UV levels were associated with a lower risk of estrogen receptor-negative (ER-) breast cancer (hazard ratio 0.73, 95% CI: 0.55–0.99).

Similar reviews and meta-analyses have shown decreased risks for incidence of **liver cancer** from prospective cohort studies (hazard ratio 0.53, 95% CI: 0.41–0.68 for subjects with the highest serum 25(OH)D compared with those with the lowest concentrations),^54^**head and neck cancer** incidence from studies assessing vitamin D intake from diet, gene polymorphisms and circulating 25(OH)D,^55^**lung cancer** (odds ratio 0.90, 95% CI: 0.83–0.97)^56^ and **ovarian cancer** from recent Mendelian randomization studies.^57^ A recent MR study that used 74 SNPs (~4% of the phenotypic variation in 25(OH)D)) found that only 2 out of 10 cancers studied had an association with vitamin D—**ovarian cancer** (OR 0.89 (95%CI 0.82–0.96) and **BCC** (OR 1.16 (95%CI 1.04–1.28) per 1 SD change).^58^ Although earlier studies of **prostate cancer** had suggested a protective effect of vitamin D, more recent evidence suggests this is not the case.^59^

Overall, the epidemiological data are supportive of reduced risks for breast, ovarian and colorectal cancer and possibly other cancers linked to solar UV exposure, although the evidence is weaker for that association being mediated by vitamin D status, particularly intake via supplementation. Several causal mechanisms mediated by vitamin D have been postulated, including reduction in cell proliferation, suppression of systemic inflammation and inhibition of angiogenesis.^60^ However, given that supplementation with vitamin D has not shown any reduction in cancer risk, it is possible that the causal pathway involves other non-vitamin D pathways, e.g. epidermal nitric oxide production from UVA.^61^ In any case, it seems likely that there is some trade-off between the risk of skin cancer from UV exposure and the benefit from reduced risk of other cancers.

### Balancing the potential benefits with the harms

Designing control measures for UV exposure of outdoor workers is complex because of the necessity to ensure that individuals understand that some exposure is necessary for good health but too much exposure can be harmful. It is prudent to seek UV exposure to ensure vitamin D sufficiency throughout the winter, but the necessary duration of exposure will vary depending on the latitude, local weather, the pattern of work, clothing worn, individual skin colour and oral vitamin D intake. For fair-skinned people in Britain it has been estimated that around 10-min exposure daily at midday would be sufficient to ensure almost all people had adequate circulating 25(OH)D throughout winter (>25 nmol/l), provided about 35% of their skin was exposed (face, hands, forearms and lower legs).^62^ The corresponding time for people with brown skin (Fitzpatrick skin type V) is about 25 min daily.^63^ Both these estimates are based on cautiously limiting UV exposure to minimize any skin cancer risk and consequently the researchers suggest that only exposing the hands and face is inadequate. However, if there are important health benefits from UV exposure, then greater exposure could be acceptable to enable workers with a smaller area of skin exposed (e.g. hands and face comprising 10% of skin area) to maintain sufficient vitamin D throughout the year.

## Discussion

New information on the exposure-response relationships between solar UV exposure and NMSC have been recently published.^37^^,^^38^ These have relied on systematic measurements of UV made with relatively low-cost instrumentation. There is an urgent need for these studies to be replicated in other contexts to substantiate the conclusions. In addition, there are limited research data on the exposure of workers in Britain and most other countries to solar UV, and further efforts to collect such data should be undertaken. Coding these data into a Job-Exposure Matrix or quantitative exposure algorithm would assist the conduct of epidemiological studies.

Clearly, management of occupational solar UV exposure is challenging: excessive exposure may cause skin cancer, whereas insufficient exposure may increase the risk of a range of other cancers and other diseases. To a large extent, the exposure of outdoor workers to solar UV is highly variable and is mediated by personal behaviour rather than just the environment, i.e. the time spent in the sun and the use of personal protective behaviours such as sunscreen and clothing.^24^ In Britain, the prevailing attitude is that sun exposure and the tan it induces are beneficial, which makes compliance with interventions to control risks low.^25^ Employers have little understanding of how best to manage sun exposure and there is no specific legislation to mandate actions. It is the responsibility of the employer to manage the environment and the behaviour of workers to ensure that people are working safely.

Ideally, we envisage workplaces taking the lead to ensure that occupational-related UV exposures provide sufficient vitamin D but not cause erythema. However, without better data on these exposures this is difficult to achieve. We suggest that employers should make more use of new low-cost methods to measure solar UV exposure to assess the exposure of their workers and manage the risks; by not relying on workers personal protective practices but changing working schedule and environment, e.g. by providing shaded areas where the work has to be undertaken. In addition, in our view, employers should undertake health surveillance to measure serum vitamin D at the end of Summer (September) to ensure that workers reach around 80.5 nmol/l to maintain 25(OH)D over 25 nmol/l levels through the winter^62^ (for 97.5% of British workers) and to identify any skin changes suggesting a risk of skin cancer; the latter already being required under health and safety law. Intervention strategies should be based on clearly articulated psychological theories to encourage workers and employers to address UV exposure issues, overcoming barriers from entrenched attitudes to exposure to sunlight, and this could involve greater use of electronic communication via text messages or social media. Strengthening the law to require employers to undertake more appropriate risk management of solar UV could provide important health benefits for workers.

There are many occupations where workers receive very low exposure to solar UV because their work is predominantly indoors, including hospital staff, office and shop workers. There may be scope to reduce the risk of colorectal, ovarian, breast and possibly other cancers for these workers by changing their work to involve some sun exposure during the day. It is likely that this could be achieved by a relatively short exposure (about 30 min daily at lunchtime, from March to September) of the forearms and lower legs.^63^

Outdoor workers in Britain with NMSC who have long-term exposure to solar UV likely have a risk more than double that of the general population. It would, in our opinion, be practicable to compensate such people under the British IIDB scheme.

## Notes

**Mark Cherrie** is a Senior Scientist at the IOM in Edinburgh, UK. He is an environmental epidemiologist, interested in how the places where we live and work shape our health across the life course. He has developed population level UV exposure metrics to understand the relationship between UV, vitamin D and several non-skeletal health outcomes.

**John Cherrie** is an Emeritus Professor of Human Health at Heriot Watt University and a Principal Scientist at the Institute of Occupational Medicine (IOM) in Edinburgh, UK. He is an exposure scientist involved in a wide range of research, including UV exposure of workers. John is a member of the British Workplace Health Expert Committee and the Industrial Injuries Advisory Council.
